# Gene Expression-Based Colorectal Cancer Prediction Using Machine Learning and SHAP Analysis

**DOI:** 10.3390/genes17010114

**Published:** 2026-01-20

**Authors:** Yulai Yin, Zhen Yang, Xueqing Li, Shuo Gong, Chen Xu

**Affiliations:** 1 School of Medicine, Nankai University, Tianjin 300071, China; 2 Department of Colorectal Surgery, Tianjin Union Medical Center, The First Affiliated Hospital of Nankai University, Tianjin 300121, China

**Keywords:** differential genes, mendelian randomization, machine learning, predictive model, colorectal cancer

## Abstract

**Objective:** To develop and validate a genetic diagnostic model for colorectal cancer (CRC). **Methods:** First, differential expression genes (DEGs) between colorectal cancer and normal groups were screened using the TCGA database. Subsequently, a two-sample Mendelian randomization analysis was performed using the eQTL genomic data from the IEU OpenGWAS database and colorectal cancer outcomes from the R12 Finnish database to identify associated genes. The intersecting genes from both methods were selected for the development and validation of the CRC genetic diagnostic model using nine machine learning algorithms: Lasso Regression, XGBoost, Gradient Boosting Machine (GBM), Generalized Linear Model (GLM), Neural Network (NN), Support Vector Machine (SVM), k-Nearest Neighbors (KNN), Random Forest (RF), and Decision Tree (DT). **Results:** A total of 3716 DEGs were identified from the TCGA database, while 121 genes were associated with CRC based on the eQTL Mendelian randomization analysis. The intersection of these two methods yielded 27 genes. Among the nine machine learning methods, XGBoost achieved the highest AUC value of 0.990. The top five genes predicted by the XGBoost method—RIF1, GDPD5, DBNDD1, RCCD1, and CLDN5—along with the five most significantly differentially expressed genes (*ASCL2*, *IFITM3*, *IFITM1*, *SMPDL3A*, and *SUCLG2*) in the GSE87211 dataset, were selected for the construction of the final colorectal cancer (CRC) genetic diagnostic model. The ROC curve analysis revealed an AUC (95% CI) of 0.9875 (0.9737–0.9875) for the training set, and 0.9601 (0.9145–0.9601) for the validation set, indicating strong predictive performance of the model. SHAP model interpretation further identified *IFITM1* and *DBNDD1* as the most influential genes in the XGBoost model, with both making positive contributions to the model’s predictions. **Conclusions:** The gene expression profile in colorectal cancer is characterized by enhanced cell proliferation, elevated metabolic activity, and immune evasion. A genetic diagnostic model constructed based on ten genes (*RIF1*, *GDPD5*, *DBNDD1*, *RCCD1*, *CLDN5*, *ASCL2*, *IFITM3*, *IFITM1*, *SMPDL3A*, and *SUCLG2*) demonstrates strong predictive performance. This model holds significant potential for the early diagnosis and intervention of colorectal cancer, contributing to the implementation of third-tier prevention strategies.

## 1. Introduction

Colorectal cancer is a malignant tumor originating in the colon or rectum [1,2]. Due to the lack of obvious symptoms and signs in the early stages, it is often diagnosed at later stages when symptoms such as rectal bleeding, bowel obstruction, abdominal distension, and weight loss become evident. Regular colonoscopy screenings are crucial for the prevention and early detection of colorectal cancer [3]. With advances in precision medicine and genomic diagnostic technologies, together with the growing demand for early detection to improve patient outcomes, current predictive models have largely focused on clinical predictors of prognosis and distant metastasis. Consequently, there is an urgent need to develop a gene-based diagnostic model capable of predicting the onset of colorectal cancer. The TCGA database provides comprehensive multi-omics data for colorectal cancer, including gene expression profiles, genomic alterations, and clinical annotations, making it an essential resource for elucidating molecular mechanisms and constructing predictive models. The IEU OpenGWAS platform aggregates a vast collection of genome-wide association study results and enables the assessment of potential causal relationships between genetic factors and colorectal cancer risk. The FinnGen project integrates large-scale genomic data with nationwide health registry information from Finland, offering a robust foundation for validating genetic risk signals and performing large-cohort predictive analyses. Meanwhile, the GEO database supplies extensive transcriptomic datasets for colorectal cancer, supporting differential expression analysis and model validation. Collectively, these databases provide a comprehensive and high-quality data framework for the present study. Therefore, this study constructs and validates a genetic diagnostic model for colorectal cancer using multiple databases, including TCGA, IEU OpenGWAS, the Finnish Database, and GEO, by employing Mendelian randomization [4,5] and nine machine learning techniques [6,7]. The goal is to provide scientific references for public health prevention at all three levels, clinical early diagnosis and treatment, as well as the promotion of precision medicine and selection of gene-targeted therapies.

## 2. Materials

This study first conducted differential gene expression analysis using the TCGA database [8]. Data was retrieved from the TCGA database (https://portal.gdc.cancer.gov/ (accessed on 20 October 2025)), where the “Cohort Builder” was used to select “TCGA” from the Program list and “TCGA-COAD” from the Project list. Next, the “Repository” menu was accessed, where the “Data Category” list was used to select “transcriptome profiling.” The filtered data was added to the Cart, and both the Cart file and Metadata file were downloaded. Specific data information can be found in Supplementary File S1.

Mendelian randomization was then employed to perform causal association analysis between eQTL genomic data [9,10] and colorectal cancer outcomes. The eQTL genomic data was sourced from the IEU OpenGWAS database [11] (https://opengwas.io/datasets/ (accessed on 20 October 2025)), and colorectal cancer outcome data was obtained from the R12 Finnish database [12] (https://r12.finngen.fi/ (accessed on 20 October 2025)).

Finally, the GEO database [13] was used to construct and validate the genetic diagnostic model. The GEO database (https://www.ncbi.nlm.nih.gov/gds (accessed on 20 October 2025)) was searched for dataset ID: GSE87211, and both the Series Matrix File and GPL file were downloaded. This dataset contains 363 samples, with 203 from the colorectal cancer group and 160 from the normal group.

## 3. Methods

### 3.1. Differential Gene Expression Analysis of the TCGA Database

The Cart data and Metadata were retrieved. These genes were converted to their respective gene symbols, and duplicates were removed. After basic data processing, genes with expression levels less than or equal to 1 were excluded. The data were then categorized into two groups: the normal group and the colorectal cancer group, with adjacent normal tissues included in the normal group. Differential gene expression analysis was performed using the rank-sum test. Genes with a log fold change (logFC) greater than 1 and a false discovery rate (FDR) less than 0.05 were selected as differentially expressed genes.

### 3.2. Mendelian Randomization Analysis of eQTL Genomics and Colorectal Cancer Outcomes

All eQTL genomic data was downloaded from the IEU OpenGWAS database as the exposure variable. To minimize the impact of linkage disequilibrium (LD), this study selected single nucleotide polymorphisms (SNPs) as instrumental variables (IVs) based on the established genome-wide significance threshold (*p* < 5 × 10^−8^, r^2^ ≤ 0.001, Hardy-Weinberg equilibrium (H-W) satisfied, genetic distance < 10,000 kb). The F-value of IVs was calculated, ensuring that only those with an F-value greater than 10 were included in the analysis to avoid bias from weak IVs. Colorectal cancer outcome data was obtained from the R12 Finnish database. A two-sample Mendelian randomization analysis was performed to assess the causal relationship between the exposure genes and colorectal cancer outcomes. The inverse variance-weighted method was applied, with results where *p* < 0.05 considered significant. Results showing heterogeneity or horizontal pleiotropy were excluded. Genes that passed these filters were considered as candidate genes associated with colorectal cancer.

### 3.3. Identification of Common Genes

In this study, we integrated GWAS-derived variants, eQTL data, and transcriptomic profiles from TCGA colorectal cancer to systematically investigate the regulatory mechanisms through which genetic variation may influence gene expression and modulate CRC susceptibility. We first performed stringent quality control on the GWAS summary statistics and harmonized all SNP coordinates to a consistent reference genome build. Significant trans-eQTL signals from colorectal and related tissues were retrieved from GTEx and other publicly available resources, and GWAS risk loci, together with their LD proxies (r^2^ > 0.8), were matched to these eQTLs using SNP-level correspondence to nominate candidate genes whose regulation might mediate CRC risk. We subsequently obtained RNA-seq data from TCGA-COAD and processed them through standard alignment, quantification, and normalization pipelines to generate expression matrices. These data were integrated with the eQTL-linked candidate genes to assess whether the regulatory directions suggested by the GWAS–eQTL associations were concordant with differential expression patterns observed between tumor and normal colorectal tissues. The differentially expressed genes from the TCGA rank-sum test and the colorectal cancer-associated genes identified through Mendelian randomization were intersected using the Venny2.1 online platform [14] (https://bioinfogp.cnb.csic.es/tools/venny/index.html (accessed on 20 October 2025)). The intersected genes from both analyses were selected as the final set for further investigation.

### 3.4. Development and Validation of the Colorectal Cancer Genetic Diagnostic Model Using Nine Machine Learning Methods Based on GEO Database

The GSE87211 dataset was retrieved, and a random seed of 12 was set. The data was split into training and validation sets with a 7:3 ratio. For the training set, predictions were developed using ten machine learning methods, including Lasso Regression, XGBoost [15], Gradient Boosting Machine (GBM), Generalized Linear Model (GLM), Neural Network (NN), Support Vector Machine (SVM), k-Nearest Neighbors (KNN), Random Forest (RF), and Decision Tree (DT), employing 10-fold cross-validation. The machine learning method yielding the highest AUC value was selected. Subsequently, the top five genes predicted by this method, along with differentially expressed genes between the colorectal cancer and normal groups in the GSE87211 dataset, were incorporated to develop a colorectal cancer gene diagnostic model. The model’s performance was then validated using the validation set.

### 3.5. SHAP Explanation of Machine Learning Models

SHAP (SHapley Additive exPlanations) is a model-agnostic explanation method [16] based on Shapley values from game theory, providing a quantitative interpretation of each feature’s contribution to a model’s predictions. SHAP values reflect the importance of each feature for a given prediction and ensure global consistency in explaining each feature’s contribution. This methodology is particularly useful for interpreting black-box models, offering insights into how individual features influence model predictions.

In this study, SHAP was employed to interpret multiple machine learning models and to explore the relationship between gene expression data and disease classification outcomes. Initially, gene expression data were read and processed, consisting of samples from two categories: “Case” and “Control.” After data preprocessing, the dataset was split into training and test sets for model training and evaluation. Several machine learning models were trained, including Lasso Regression, XGBoost, Gradient Boosting Machine (GBM), Generalized Linear Model (GLM), Neural Network (NN), Support Vector Machine (SVM), k-Nearest Neighbors (KNN), Random Forest (RF), and Decision Tree (DT). Model performance was evaluated using 5-fold cross-validation, with the area under the curve (AUC) serving as the primary evaluation metric. Based on a comparison of AUC values, the best-performing model was selected as the basis for SHAP analysis.

After selecting the optimal model, SHAP values were computed using the kernelshap package. The goal of calculating SHAP values was to understand the contribution of each gene (feature) to the model’s predictions. During the computation, a prediction function was defined using the training dataset to output the predicted probability of each sample belonging to the “Case” category. This function was applied to the training data, yielding SHAP values for each feature. Finally, visualizations such as feature importance bar plots, importance beeswarm plots, dependence plots, waterfall plots, and force plots were generated to present the SHAP values and interpret the contributions of the features.

## 4. Results

### 4.1. Gene Expression in Colorectal Cancer: Trends Toward Cell Proliferation, High Metabolism, and Immune Escape

**Figure 1:**
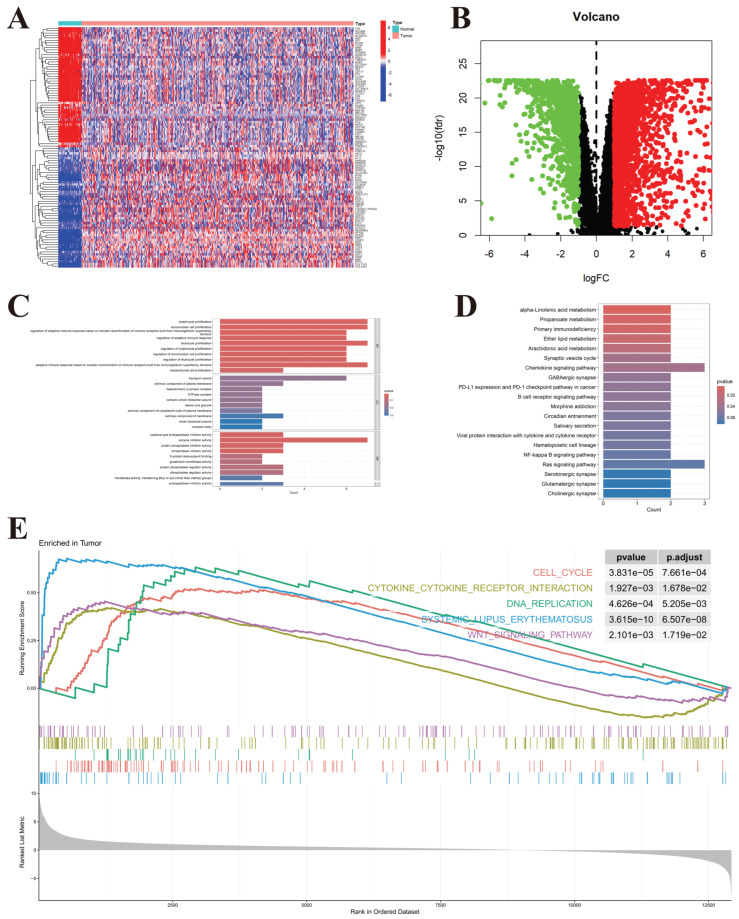
Pathway Enrichment of Differential Genes in Colorectal Cancer. (**A**) Heatmap of the top 50 differentially expressed genes in colorectal cancer, showing the gene expression patterns between the colorectal cancer and normal groups. (**B**) Volcano plot of differentially expressed genes in colorectal cancer, highlighting genes with significant expression changes. Red dots represent upregulated genes, and green dots represent downregulated genes. The x-axis denotes the log fold change (logFC), while the y-axis represents statistical significance (−log10 of *p*-value). (**C**) GO enrichment analysis bar plot of the differentially expressed genes in colorectal cancer. The plot shows the enrichment of the genes in biological processes (BP), cellular components (CC), and molecular functions (MF). (**D**) KEGG pathway enrichment analysis bar plot of the differentially expressed genes in colorectal cancer, showing the significant pathways that are enriched in the dataset. (**E**) GSEA enrichment analysis plot for colorectal cancer, demonstrating the significant pathways enriched in tumor samples. The upper-right corner of the plot displays the *p*-value and the corrected *p*-value after statistical adjustment. “Enriched in Tumor” denotes that the gene sets exhibit higher enrichment in tumor samples relative to normal tissues. “Running Enrichment Score” refers to the cumulative score computed along the ranked gene list in the GSEA analysis, where higher values indicate stronger overrepresentation of genes from the corresponding gene set.

A heatmap of the top 50 differentially expressed genes was generated, highlighting that genes such as SPRR family members, MUC6, KRT family genes, SLC26A9, MMP7, PRSS56, and SFRP1 were highly expressed in the colorectal cancer group. These highly expressed genes are predominantly associated with tumor cell proliferation, invasiveness, immune escape, and extracellular matrix remodeling (Figure 1A,B). GO (Gene Ontology) and KEGG (Kyoto Encyclopedia of Genes and Genomes) enrichment analyses were performed on the differential genes. GO enrichment analysis revealed that these genes were primarily enriched in pathways related to lymphocyte proliferation and monocyte proliferation, suggesting a potential link to immune microenvironment regulation (Figure 1C). KEGG pathway enrichment analysis showed that the differential genes were enriched in pathways related to α-linolenic acid metabolism and arachidonic acid metabolism, indicating associations with anti-inflammatory effects, immune regulation, and angiogenesis (Figure 1D). Furthermore, GSEA (Gene Set Enrichment Analysis) revealed significant enrichment of the differential genes in five signaling pathways in tumor samples: cell cycle, DNA replication, cytokine-cytokine receptor interaction, systemic lupus erythematosus (SLE), and Wnt signaling pathways. These results suggest that the differential genes play a role in cell proliferation, DNA replication, tumor invasion, migration, and autoimmune responses (Figure 1E).

### 4.2. Differential Expression of Intersection Genes from TCGA and MR Analysis Focused on Cell Proliferation

**Figure 2:**
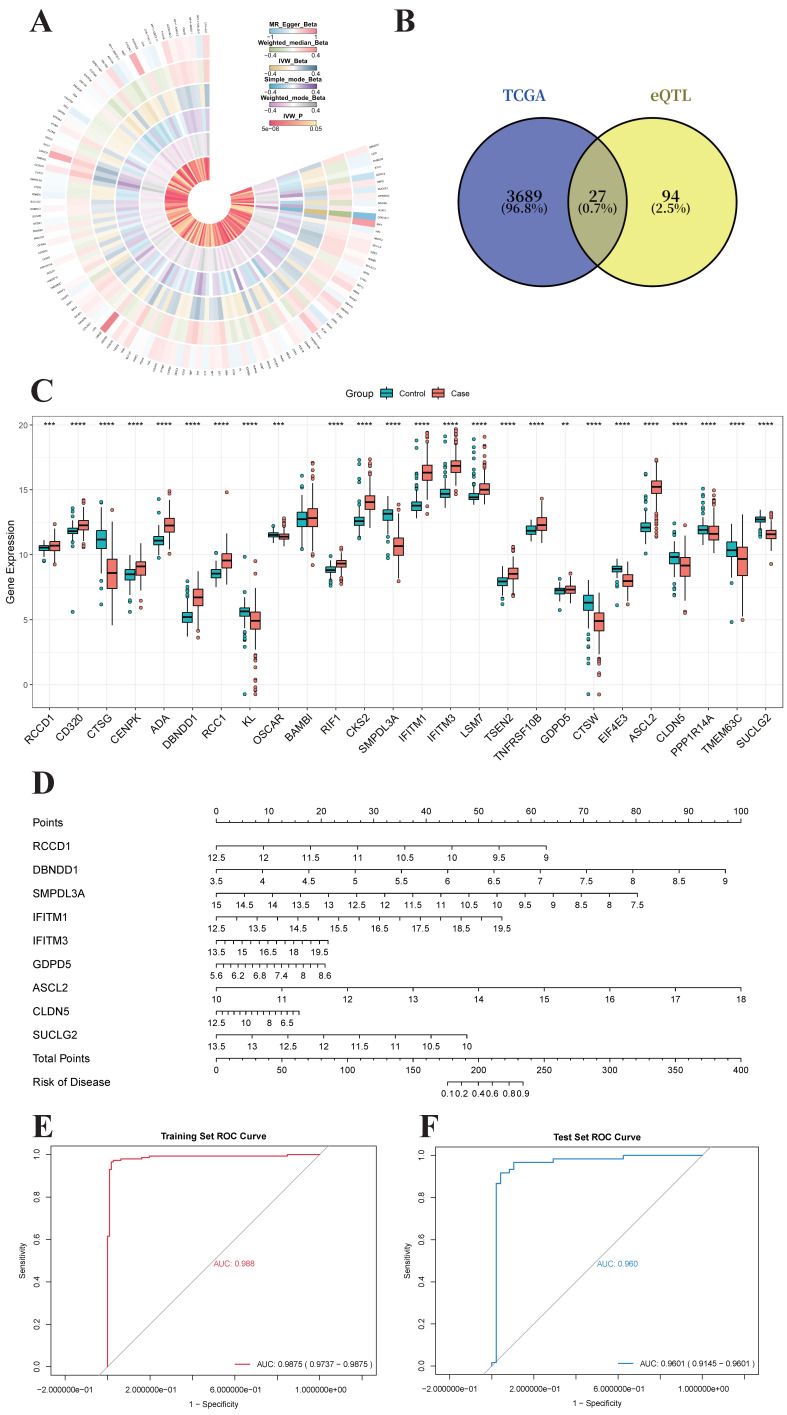
Mendelian Randomization Results and Development and Validation of the Genetic Diagnostic Model. (**A**) Circular heatmap of Mendelian randomization (MR) analysis results. The outermost circle represents the positive genes, followed by the Beta values from five different MR methods. The innermost circle represents the *p*-values from the Inverse Variance Weighted (IVW) method. (**B**) Venn diagram showing the intersection of genes between TCGA and MR analyses. The 27 common genes identified from both datasets are displayed. (**C**) Box plot of differential expression of the 27 intersecting genes, comparing expression levels between the colorectal cancer and normal groups. ** denotes *p* < 0.01, *** denotes *p* < 0.001, and **** denotes *p* < 0.0001. (**D**) The nomogram for the colorectal cancer genetic diagnostic model was developed using genes selected from Mendelian randomization (MR) and TCGA analyses. The numbers displayed above each gene (*RIF1*, *GDPD5*, *DBNDD1*, *RCCD1*, *CLDN5*, *ASCL2*, *IFITM3*, *IFITM1*, *SMPDL3A*, and *SUCLG2*) represent the gene expression levels incorporated as predictors in the model. Each expression value is mapped to a corresponding point on the top “Points” axis. The point values for all genes are then summed to yield the “Total Points,” which are subsequently converted into the predicted probability of disease on the “Risk of Disease” axis. (**E**) ROC curve for the training set of the colorectal cancer genetic diagnostic model. The bottom-right corner of the plot displays the corresponding AUC value and 95% confidence interval (CI). (**F**) ROC curve for the validation set of the colorectal cancer genetic diagnostic model. The bottom-right corner of the plot shows the corresponding AUC value and 95% confidence interval (CI).

Mendelian randomization (MR) analysis identified 121 genes associated with colorectal cancer (Figure 2A), while differential gene expression analysis from TCGA revealed 3716 genes. A Venn diagram demonstrated 27 common genes between the two datasets (Figure 2B). Differential expression analysis of these 27 intersecting genes in the GSE87211 dataset from GEO revealed significant expression differences between tumor and normal groups for 26 genes, with *ASCL2*, *IFITM3*, *IFITM1*, *SMPDL3A*, and *SUCLG2* being the five most significantly differentially expressed (Figure 2C). These five genes may play pivotal roles in tumor proliferation, metastasis, and drug resistance by regulating cell proliferation, repair mechanisms, and signaling pathways.

### 4.3. Development and Validation of the Colorectal Cancer Genetic Diagnostic Model Using Nine Machine Learning Methods

The GSE87211 dataset comprises 363 samples, with 203 from the colorectal cancer group and 160 from the normal group. The dataset was split into training and validation sets in a 7:3 ratio, using a random seed of 12 to ensure reproducibility. Nine machine learning methods—Lasso regression, XGBoost, Gradient Boosting Machine (GBM), Generalized Linear Model (GLM), Neural Network (NN), Support Vector Machine (SVM), k-Nearest Neighbors (KNN), Random Forest (RF), and Decision Tree (DT)—were employed to develop predictive models. The results indicated that the XGBoost method achieved the highest AUC value of 0.990. Consequently, the top five genes predicted by XGBoost (*RIF1*, *GDPD5*, *DBNDD1*, *RCCD1*, and *CLDN5*), along with five intersection genes (*ASCL2*, *IFITM3*, *IFITM1*, *SMPDL3A*, and *SUCLG2*) that showed differential expression in the GSE87211 dataset, were used to construct the colorectal cancer genetic diagnostic model (Figure 2D). ROC curves were generated for both the training and validation sets of the new model. The results revealed an AUC value of 0.9875 (95% CI: 0.9737–0.9875) for the training set and 0.9601 (95% CI: 0.9145–0.9601) for the validation set, demonstrating strong predictive performance (Figure 2E,F).

### 4.4. SHAP Explanation of the XGBoost Model

**Figure 3:**
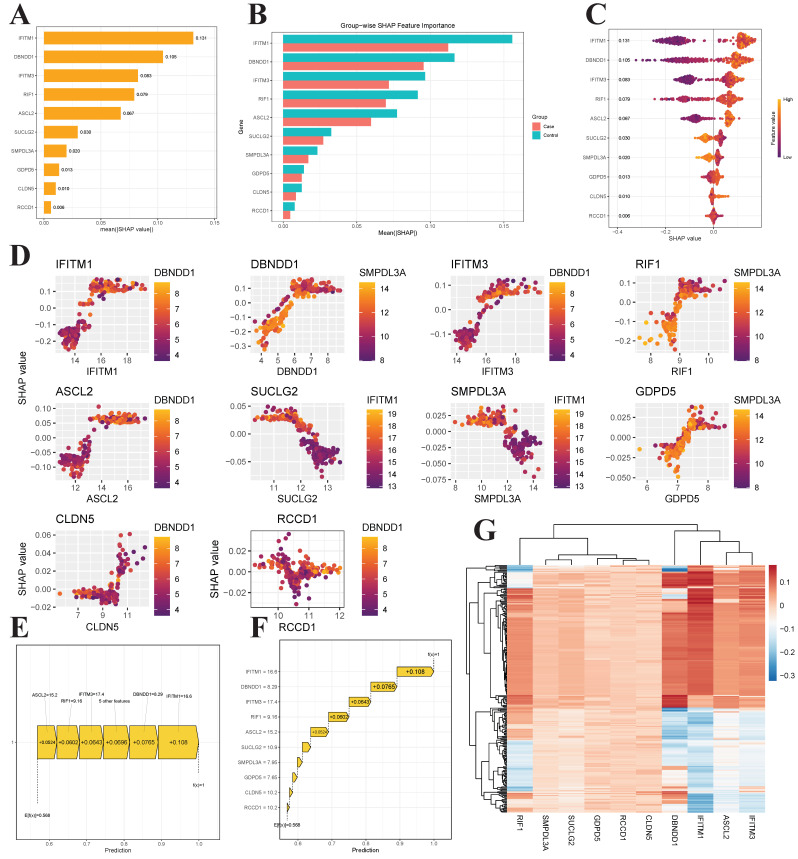
SHAP Explanation of the XGBoost Model. (**A**) Bar Plot of Gene Importance. (**B**) Grouped Gene Importance Bar Plot. (**C**) Gene Importance Beeswarm Plot. (**D**) Scatter Plot of Gene Expression and SHAP Values. (**E**) Gene Force Plot. (**F**) Gene Waterfall Plot. (**G**) Heatmap of Gene SHAP Values.

Based on the maximum AUC value, the most reliable model among the nine machine learning methods was selected as XGBoost. Through SHAP analysis of the XGBoost model, we revealed the contribution of each feature in gene expression data to colorectal cancer (CRC) prediction. The SHAP value quantifies the impact of a given feature on the model’s prediction by comparing the model outputs with and without that feature across all possible feature subsets. By averaging these marginal prediction differences, SHAP provides an estimate of the overall positive or negative contribution of the feature to the final prediction. Initial SHAP analysis indicated that the *IFITM1* gene had the greatest contribution to the model’s prediction, with an average SHAP value of 0.131, signifying its most significant impact on the prediction outcome. Other genes, such as *DBNDD1* and *IFITM3*, also exhibited high importance, contributing average SHAP values of 0.105 and 0.083, respectively (Figure 3A). Further group-based analysis (Case vs. Control) revealed that *IFITM1* had higher SHAP values in the control group, suggesting that this gene plays a crucial role in disease prediction (Figure 3B).

Through importance beeswarm plots and scatter plot matrices (Figure 3C,D), we observed the distribution of SHAP values for differentially expressed genes, where the expression levels of *SUCLG2*, *SMPDL3A*, and *RCCD1* were negatively correlated with their SHAP values, while the expression of other genes showed a positive correlation with their SHAP values. Waterfall and bar plots (Figure 3E,F) further confirmed the major contributions of *IFITM1*, *DBNDD1*, and *IFITM3* genes to the model’s prediction, with *IFITM1* and *DBNDD1* showing particularly strong positive contributions, increasing the prediction probability by 0.108 and 0.0765, respectively. The SHAP value heatmap highlighted the significant contributions of *IFITM1*, *DBNDD1*, and *IFITM3* to the model (Figure 3G).

## 5. Discussion

Colorectal cancer (CRC) is a disease of significant concern due to its poor prognosis in advanced stages and the high likelihood of liver metastasis. According to the Global Disease Burden 2021 data, the age-standardized incidence rate of colorectal cancer globally in 2021 was 25.607 per 100,000 people, while the age-standardized prevalence reached 134.837 per 100,000 people [17]. These statistics highlight the heavy disease burden of colorectal cancer, making the development of effective screening and prediction methods for early detection crucial. This would significantly aid in promoting tertiary prevention, early diagnosis, and treatment, improving long-term patient outcomes, and alleviating the overall disease burden [18,19]. Despite the critical need for such advancements, a comprehensive genetic diagnostic model for CRC that integrates data from multiple databases and applies advanced machine learning methods remains underdeveloped. This study addresses this gap by constructing and validating a robust genetic diagnostic model for CRC. The model leverages data from TCGA, IEU OpenGWAS, the R12 Finnish database, and the GEO database, employing rank-sum tests, two-sample Mendelian randomization (MR), and nine machine learning techniques.

This study also identified ten genes—*RIF1*, *GDPD5*, *DBNDD1*, *RCCD1*, *CLDN5*, *ASCL2*, *IFITM3*, *IFITM1*, *SMPDL3A*, and *SUCLG2*—as significant contributors to the genetic diagnostic model. Among these, *IFITM1*, *DBNDD1*, and *IFITM3* were the top three genes contributing to the CRC genetic prediction model. Rohan Gupta et al. [20] demonstrated that AQP9 and *IFITM1* are driving factors in immune infiltration and tumor progression in IBD-related colorectal cancer. Their study also showed that key IBD genes exhibit genomic alterations, such as *AQP9* (2.3%), *IFITM1* (1%), and *ITGA5* (3%). Notably, *AQP9* and *IFITM1*, as inflammation-related features, displayed higher carcinogenic potential. This is consistent with our findings that higher expression of *IFITM1* is associated with an increased risk of colorectal cancer. Xiaomin Qi et al. [21] showed that elevated levels of DBNDD1 and growth differentiation factor 15 (GDF15) in colorectal cancer promote sustained activation of NF-κB (RELA) through a DBNDD1-dependent mechanism. Silencing DBNDD1 inhibited cell proliferation, migration, and invasion (in DLD1/HCT116 cells) while reducing GDF15 expression and decreasing p-NF-κB p65–p-I-κB signaling. Moreover, DBNDD1 knockdown slowed tumor growth in vivo, highlighting the DBNDD1–GDF15–NF-κB signaling pathway as a driver of colorectal cancer pathogenesis. This potential mechanism also corroborates our findings that higher DBNDD1 expression is linked to an increased risk of colorectal cancer. *IFITM3*, another important gene in the IFITM family, has been implicated in colorectal tumorigenesis. Pauline Andreu et al. [22] revealed the association between IFITM family genes and colorectal tumor development. Their research demonstrated that IFITM gene expression is rapidly induced following β-chain protein signaling activation. Large-scale analysis of human tumors indicated significant upregulation of IFITM gene expression in colorectal cancer, suggesting that IFITM family genes may serve as valuable diagnostic tools for these tumors. This aligns with our findings, where both *IFITM1* and *IFITM3* from the IFITM family contribute significantly to the genetic risk prediction of CRC.

In our study, TCGA analysis revealed that genes such as those from the SPRR family, MUC6, KRT family, *SLC26A9*, *MMP7*, *PRSS56*, and *SFRP1* were highly expressed in the colorectal cancer group. These genes are mostly involved in tumor cell proliferation, invasiveness, immune evasion, and extracellular matrix remodeling. Studies by Suwen Ou et al. [23] found that MMP7 is associated with colorectal cancer metastasis and poor prognosis, while Roopali Roy et al. [24] discovered that MMP family members mediate cell proliferation, migration, invasion, differentiation, apoptosis, inflammation, and angiogenesis by degrading ECM proteins such as collagen type I and IV, laminin, fibronectin, fibrinogen, and proteoglycans, promoting both physiological and pathological processes. Additionally, Dandan Li’s research [25] indicated that the PRSS56 gene promotes colorectal cancer progression via the PI3K/AKT signaling axis.

This study proposes a genetic-based diagnostic model for CRC, a significant advancement over existing models that largely rely on histopathology, radiomics, or immune scores. Renjie Wang et al. [26] developed a prognostic model for pulmonary metastasis of colorectal cancer integrating pathomics, radiomics, and immune scoring, demonstrating substantial clinical utility. Their study showed that the predicted pathomics and radiomics scores were negatively correlated with immune scores, and that all three served as independent prognostic factors for both OS and DFS. Importantly, their work focused primarily on forecasting postoperative outcomes such as OS and DFS in patients with colorectal cancer, whereas the present study aims to predict the risk of colorectal cancer onset. In addition, while the predictive features in Wang et al.’s model centered on immune scoring, pathomics, and radiomics, our study concentrates on gene-expression profiles, representing a fundamental methodological distinction. Similarly, Rui Cao et al. [27] proposed a pathology-based model to predict microsatellite instability (MSI) in colorectal cancer, offering meaningful guidance for subsequent therapeutic decision-making. Their findings demonstrated that deep-learning models applied to histopathological images can robustly infer MSI status and generalize effectively to external patient cohorts. However, their work focused on predicting MSI from histological images, with predictive factors and clinical endpoints that differ markedly from those of the present study. Collectively, existing models based on genomic features for the diagnosis of colorectal cancer remain limited. The present study therefore provides a valuable complement to current research efforts in this field.

In genetic studies, confounders, batch effects, and platform differences can introduce significant biases. To minimize these effects, we ensured harmonization of the data from TCGA, IEU OpenGWAS, the R12 Finnish database, and the GEO database by applying consistent preprocessing steps, including normalization and scaling. We also addressed batch effects using statistical methods such as ComBat, which adjusts for batch-specific variations. Furthermore, differences in platforms (e.g., RNA-seq vs. microarray) can introduce variability in gene expression data. We employed platform-specific quality control measures to minimize such discrepancies, ensuring that the results were not unduly influenced by technological differences.

While this study offers significant advancements, several limitations should be noted:

Colon vs. Rectal Cancer Subgroups: Our model does not differentiate between colon cancer and rectal cancer, which may have distinct molecular features and prognostic factors. Future studies should aim to develop subgroup-specific models to improve diagnostic accuracy.

Microsatellite Instability: The current model does not incorporate microsatellite status, a critical factor for prognosis and treatment decisions in CRC. Incorporating microsatellite instability status into the diagnostic model would enhance its clinical applicability.

External Validation: Although we validated the model using data from several publicly available databases, an important limitation is the lack of external validation with independent, large-scale cohort data. Future validation efforts should aim to apply the model to external datasets to assess its generalizability across different populations.

Model Evaluation: The current study did not extensively evaluate model performance using calibration curves or Decision Curve Analysis (DCA), which are crucial for assessing clinical utility. Future work should incorporate these metrics to provide a more comprehensive evaluation of the model’s performance in real-world clinical settings.

Overfitting Risk: The use of multiple machine learning techniques increases the complexity of the model, raising the risk of overfitting. Cross-validation techniques and external datasets are needed to ensure the model’s robustness and reliability.

Population Heterogeneity: Due to the diverse ethnic composition represented in TCGA, whereas FinnGen predominantly comprises individuals of Finnish descent, there may be differences in genetic structure, mutation profiles, and cancer susceptibility between the Finnish population and the multi-ethnic cohort in TCGA. These population-level differences could potentially affect the generalizability of gene overlap results across the two databases.

## 6. Conclusions

This study reveals that differentially expressed genes in colorectal cancer are primarily associated with cell proliferation, high metabolism, and immune evasion, with cell proliferation being the dominant trend. The genetic diagnostic model developed based on ten genes—*RIF1*, *GDPD5*, *DBNDD1*, *RCCD1*, *CLDN5*, *ASCL2*, *IFITM3*, *IFITM1*, *SMPDL3A*, and *SUCLG2*—demonstrates strong predictive performance. This model holds significant implications for tertiary prevention, as well as for the early diagnosis and treatment of colorectal cancer.

## Data Availability

The data used in this study were all obtained from publicly available databases (The Cancer Genome Atlas (TCGA) database: https://portal.gdc.cancer.gov/ (accessed on 20 October 2025); IEU OpenGWAS database: https://opengwas.io/datasets/ (accessed on 20 October 2025); R12 Finnish database: https://r12.finngen.fi/ (accessed on 20 October 2025); Gene Expression Omnibus (GEO) database: GSE87211, https://www.ncbi.nlm.nih.gov/gds (accessed on 20 October 2025)), and the original studies have received ethical approval. Therefore, ethical review by an institutional review board (IRB) was not required for this study.

## Supplementary Materials

The following supporting information can be downloaded at: https://www.mdpi.com/article/10.3390/genes17010114/s1.
